# Enhancement of Flame Retardancy and Mechanical Properties of Polylactic Acid with a Biodegradable Fire-Retardant Filler System Based on Bamboo Charcoal

**DOI:** 10.3390/polym13132167

**Published:** 2021-06-30

**Authors:** Wenzhu Li, Liang Zhang, Weisheng Chai, Ningning Yin, Kate Semple, Lu Li, Wenbiao Zhang, Chunping Dai

**Affiliations:** 1 Zhejiang Provincial Collaborative Innovation Center for Bamboo Resources and High-Efficiency Utilization, College of Chemistry and Materials Engineering, Zhejiang A&F University, Hangzhou 311300, China; lwz@zafu.edu.cn (W.L.); chaselz520103@163.com (L.Z.); shinkuu1995@163.com (W.C.); ynn8520@163.com (N.Y.); lilu@zafu.edu.cn (L.L.); 2 Department of Wood Science, Faculty of Forestry, University of British Columbia, 2900-2424 Main Mall, Vancouver, BC V6T 1Z4, Canada; katherine.semple@ubc.ca

**Keywords:** bamboo charcoal, chitosan, polylactic acid, flame retardant, cooperative system

## Abstract

A cooperative flame-retardant system based on natural intumescent-grafted bamboo charcoal (BC) and chitosan (CS) was developed for polylactic acid (PLA) with improved flame retardancy and minimal decline in strength properties. Chitosan (CS) as an adhesion promoter improved the interfacial compatibility between graft-modified bamboo charcoal (BC-m) and PLA leading to enhanced tensile properties by 11.11% and 8.42%, respectively for tensile strength and modulus. At 3 wt.% CS and 30 wt.% BC-m, the crystallinity of the composite increased to 38.92%, or 43 times that of pure PLA (0.9%). CS promotes the reorganization of the internal crystal structure. Thermogravimetric analysis showed significantly improved material retention of PLA composites in nitrogen and air atmosphere. Residue rate for 5 wt.% CS and 30 wt.% BC-m was 29.42% which is 55.1% higher than the theoretical value of 18.97%. Flammability tests (limiting oxygen index-LOI and UL-94) indicated significantly improved flame retardancy and evidence of cooperation between CS and BC-m, with calculated cooperative effectiveness index(Ce) >1. From CONE tests, the peak heat release rate (pHRR) and total heat release (THR) were reduced by 26.9% and 30.5%, respectively, for 3% CS + 20% BC-m in PLA compared with adding 20% BC-m alone. Analysis of carbon residue morphology, chemical elements and structure suggest CS and BC-m form a more stable char containing pyrophosphate. This char provides heat insulation to inhibit complete polymer pyrolysis, resulting in improved flame retardancy of PLA composites. Optimal mix may be recommended at 20% BC-m + 3% CS to balance compatibility, composite strength properties and flame retardance.

## 1. Introduction

Universal dependence on petroleum-based plastics has created enormous land and sea pollution problems prompting research and development of new bio-based and biodegradable polymers [1,2]. Polylactic acid (PLA) is an example, with excellent mechanical properties, high processability, and potential for regeneration and biodegradability [3,4], and is today widely used in packaging, the medical industry, and 3D printing [5,6,7,8]. However, its inherent biodegradability and fermented biomass-derived carbon-hydrogen composition give PLA low heat and melt resistance when burned [9,10,11], causing flame spread due to melting and dripping [12]. For this reason, PLA does not meet the UL-94 V-1 rating or higher required to mitigate fire hazard, limiting its applications [13].

Various strategies have been proposed to improve the flame retardancy of PLA [14,15]. Previous work by authors include modification with aluminum hypophosphite (AHP) [16,17] and bamboo charcoal (BC) [18]. The flame-retardant performance was improved, but mechanical properties decreased and combustion produced toxic phosphene gas (PH_3_). While BC+AHP was effective in PLA, it is not commercially viable as a “green” packaging material. Renewable, bio-based, non- or low-toxic and low-smoke intumescent flame retardants (IFR) are being developed [7,19]. Biomass materials, such as starch, lignin, chitosan (CS), and sodium alginate [20,21], have been investigated as flame-retardant charring agents in polymers. A new bio-based flame retardant (defined as LHP) containing hydroxyapatite and lignocellulose in a ratio of LHP to APP of 1:3 at 10 wt.% addition is effective [14]. The peak heat release value in cone calorimeter combustion (CONE) tests decreased from 365 kW/m^2^ to 250 kW/m^2^. Beta-cyclodextrin (β–CD) and poly (propylene glycol) (PPG) have been used to improve the flame retardancy of PLA with ammonium polyphosphate (APP) and melamine (MA) [22]. A limiting oxygen index (LOI) of 34 vol% and a UL-94 V-0 vertical flame test rating were both achieved. Besides, the addition of 1–5 wt.% starch was also used to further increase the LOI to 30.6% and improve the anti-dripping properties of PLA-based foams [23]. However, these additives require a high loading for efficacy which significantly reduces the mechanical properties of the materials [24,25].

Chitosan (CS), the fully or partially deacetylated form of chitin, is one of the very few natural polymers that has a primary amino group along its main chain [26]. It has excellent biocompatibility, film-forming properties, and different viscosity states, and is therefore used as the reinforcing phase in polymer composites [27,28,29]. CS on its own has little flame retardancy with a carbohydrate structure that does not promote carbonization; however, its matrix can be modified with inorganic nanofillers to give unique properties. For example, adding halloysite can significantly raise the ignition temperature to around 150 °C, although a large number of active layers are needed [30,31]. Though the flame retardancy of CS can be increased by the formation of a carbon layer in the presence of phosphoric acid [21,32,33], phosphorylated chitosan (CS-P) often displays poor thermal stability, which limits its application in many polymers [34]. However CS in different states may be used to form a cooperative system with a char-forming intumescent graft-modified BC, developed by Zhang et al. (2021) [18]. BC has a very high specific surface area and a rich, irregular pore structure that is effectively graft modified with phosphorous and nitrogen compounds to produce an effective flame retardant that mixes well with PLA. The objective of this work was to develop and test a non-toxic, more effective, and biodegradable flame-retardant system based on combining BC-m with CS with minimal negative impacts on the mechanical properties of polylactic acid.

## 2. Materials and Methods

### 2.1. Materials

PLA resin was purchased from Cargill Dow Inc (4032D, Nature Works Co. Ltd., Blair, NE, USA) with a density of 1.24 g/cm^3^ and melting point of 160 °C. The glass transition temperature and the crystallization peak temperature of the polymer are 57.8 °C and 150–170 °C, respectively. BC was supplied by Zhejiang Anji Huasen Bamboo Charcoal Products Co., Ltd. (Zhejiang, China). The product was carbonized at 700 to 800 °C and ground to 100 to 250 mesh particle size. CS was supplied by Macklin Biochemical Co., Ltd., (Shanghai, China), with viscosity of 200–400 mPa.s.

Chemical reagents used for graft modification of BC were: hydrogen peroxide (H_2_O_2_) at 30% conc. (Yonghua Chemical Reagent Co., Ltd., Jiangsu, China), phosphoric acid (PA) at 85% conc. and urea at 99% purity (both from Sinopharm Chemical Reagent Co., Ltd., Shanghai, China), and ammonium hydroxide solution at 3% conc. and 0.91 g/mL density at 20 °C (Macklin Biochemical Co., Ltd., Shanghai, China).

### 2.2. Preparation

#### 2.2.1. Grafting Modification of BC

As described in previous work [18], two batches of 30% conc. aqueous H_2_O_2_ solution were used to treat BC (which was thoroughly washed and dried) in succession. Using a BC to H_2_O_2_ of 1:3, the BC was treated for 8 h. After filtration, H_2_O_2_ was added again, followed by 1 h of microwave irradiation at 200 W, and oven drying at 90 °C overnight to produce a treated BC intermediate termed “BC-o”.

The BC-o powder was reacted with PA at 85% conc. using a 1:1 ratio to BC-o and stirring at 80 °C for 30 min, then gradually heated further to 100 °C and maintained for 1 h. Urea dispersed in deionized water was added for a PA:urea ratio of 1:1.8, with 3% ammonia used to adjust the pH of the mixture to between 8 and 10. The mixture was heated slowly to 120 °C for 30 min and then gradually increased to 160 °C, which was then cured in an oven at 200 °C for 6 h. The hardened foam, termed “BC-m”, was ground to 150–250 mesh and washed several times with a hot ethanol solution and dried at 90 °C for 2 h.

#### 2.2.2. Preparation of Flame-Retardant PLA Composites

PLA, CS, and BC-m powders were dried at 80 °C overnight. A twin-roll mixing mill (XK-160, Wuhan, China) was used to compound them together at 180 °C. The screw was run at 30 rpm. Different mix ratios of PLA, CS, and BC-m (shown in Table 1) were prepared.

**Figure 1:**
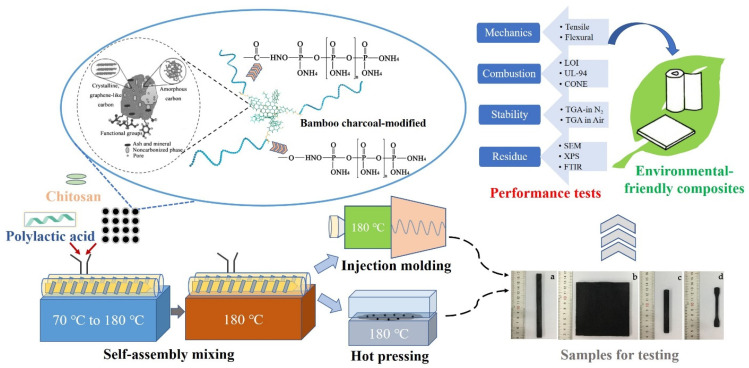
Schematic diagram of composite preparation and testing.

The compounded, extruded mixes were cut into pellets and re-mixed in the mill until a visually good dispersion was achieved. The compounded pellets were hot-pressed (180 °C) into sheets with under 10 MPa pressure for 10 min for combustion performance tests. Pellets were also injection molded into splines at 175 °C and 6 MPa pressure, which were used for mechanical tests of tensile and flexural properties. A schematic diagram of the preparation and testing processes is shown in Figure 1.

### 2.3. Measurement and Characterization

#### 2.3.1. Characterization of PLA Composites

The fracture surface morphology of PLA and the blends with BC-m and CS were observed by a scanning electron microscope (SEM; TM3030, Hitachi, Japan) at an accelerating voltage of 15 kV. Samples were conductive coated by gold sputter coating for 90 s at a current of 15 mA to get a conductive layer.

An X-ray diffractometer (XRD6000, Shimadzu Co., Ltd., Kyoto, Japan) was used to detect changes in the crystal structure. The profiles were analyzed by Cu Kα (0.15406 nm) radiation under a tube voltage of 40 kV. The tube current was set at 30 mA, and the scanning range was 3°–40° (2θ) at a rate of 2°/min.

Differential scanning calorimetry (DSC) tests were conducted using DSC-500B (YANJIN Scientific Instrument Co., Ltd., Shanghai, China) under nitrogen at a flow rate of 50 mL/min. Samples of 5 mg were weighed and sealed in a tared aluminum pan with an empty pan used as the reference. Samples were first heated from 25 to 220 °C at a rate of 10 °C/min^−1^, kept for 5 min to eliminate the thermal history, then cooled down to 25 °C at a rate of 10 °C/min. The second heating curve was recorded at a heating rate of 10 °C/min, showing the melting process. Crystallinity (*X*_c_) was estimated according to Equation (1),

(1) $$X_{c}(\%)=\frac{△H_{C}}{△H_{0}\cdot X_{\mathrm{PLA}}}\;\times \;100\%$$

where $△H_{C}$ refers to the crystallization enthalpy; $△H_{0}$ refers to the enthalpy value during 100% crystallization of PLA, which is 93.6 J/g [35]; *X*_PLA_ refers to the weight ratio of PLA in the FPLA composite blends.

#### 2.3.2. Mechanical Properties

The mechanical properties (tensile strength and modulus, MOR, MOE) of the composites were tested using a microcomputer-controlled electronic universal testing machine (CMT610, MTS Industrial System (China) Co., Ltd., Shanghai, China) according to the Chinese National Testing Standard GB/T 1040-2006 (for tensile properties) and GB/T 9341-2008 (for flexural properties). For the tensile test, the width and thickness of the narrow stress zone in specimens were 5 and 2 mm, respectively. The gauge length was set to 60 mm and loading speed was 2 mm/min. The flexural test specimen dimensions were 80 × 10 × 4 mm^3^ with load span of 60 mm and loading speed of 2 mm/min.

#### 2.3.3. Thermal Behavior

Thermal gravimetric analysis (TGA) was carried out using a thermo-gravimetric analyzer (F1, NETZSCH Co., Ltd., Selbu, Germany) under nitrogen and air atmosphere. Approximately 5–10 mg of material was weighed and assayed in an aluminum crucible and tested over a temperature range of 50 to 700 °C with a heating rate of 10 °C/min under a gas flow of 20 mL/min. An empty crucible was used as a reference.

#### 2.3.4. Combustion Properties Tests

The limiting oxygen index (LOI), expressed as vol.% minimum concentration of oxygen required to initiate combustion, was measured using a JF-3 oxygen index meter (Jiangning Analysis Instrument Company, Nanjing, Jiangsu, China) according to ISO 4589-2:2017 [36]. The dimensions of the test specimens were 100 ×10 × 4 mm^3^.

The Underwriters Laboratory (UL-94) vertical flame test was carried out using a 5402-type vertical burn test instrument (Suzhou Yangyi Woerqi Detection Technology Co., Ltd., Nanjing, Jiangsu, China) with specimens measuring 130 ×13 × 3 mm^3^. The test parameters and ratings are defined in ATSM D3801-10 [37].

Cone calorimeter combustion (CONE) tests were carried out using a cone calorimeter (FTT0007, Fire Testing Technology Co., Ltd., East Grinstead, UK) according to ISO 5660-1 [38]. The specimen measuring 100 × 100 × 4 mm^3^ was placed horizontally in a bed of aluminum foil and was heated under the combustion hood in which the predetermined test height was 25 cm. Using an external heat flux of 35 kW/m^2^, the material was ignited and the relevant combustion performance indices recorded until combustion was complete. Combustion indices are the average of 3 test specimens per group.

#### 2.3.5. Residue Analysis

All remaining carbonized residues from the CONE tests were collected and imaged to further analyze the surface morphology using a scanning electron microscope (SEM) at an accelerating voltage of 15 kV. Specimens were sputter coated with 24 ct gold for 90 s at a current of 15 mA.

Fourier transform infrared spectroscopy (FTIR) was used to detect the changes in chemical groups and structure of the residues using a Nicolet 6700FT-IR spectrophotometer with KBr slice. The transmission mode was used and the wave number range was set from 400 cm^−1^ to 4000 cm^−1^.

X-ray photoelectron spectroscopy (XPS) was used to investigate the residual element contents of samples. This was carried out using a Thermo Scientific^TM^ K-Alpha^TM+^ spectrometer (Thermo Fisher, Scientific Inc, Waltham, MA, USA) equipped with a monochromatic Al Kα X-ray source (1486.6 eV) operating at 100 W. Samples were analyzed under vacuum (*p* < 10^−8^ mbar) with a pass energy of 150 eV (survey scans) or 25 eV (high-resolution scans). All peaks were calibrated with C1s peak binding energy at 284.8 eV for adventitious carbon. The experimental peaks were fitted using Avantage software.

## 3. Results

### 3.1. Characterization of PLA and FPLA Composites

#### 3.1.1. Micromorphology and Dispersibility

**Figure 2:**
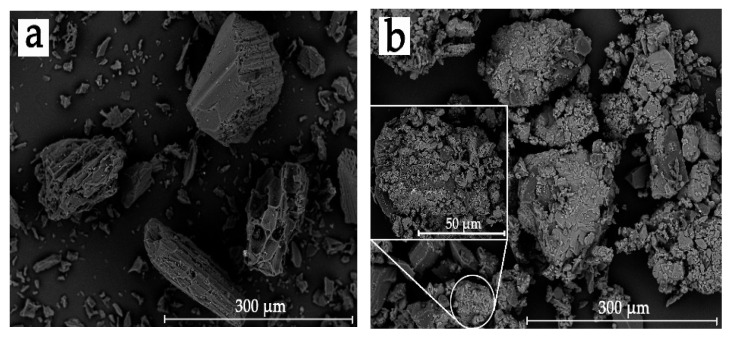
SEM images of surface morphology: (**a**) BC and (**b**) BC-m.

The surface morphology of BC and BC-m are shown in Figure 2. The original pore structure of the bamboo tissue can be clearly seen on the surface of BC particles (Figure 2a), while the surface of BC-m became amorphous (Figure 2b). There was little difference in particle size of BC before and after modification, which was mainly between 50 and 100 μm.

**Figure 3:**
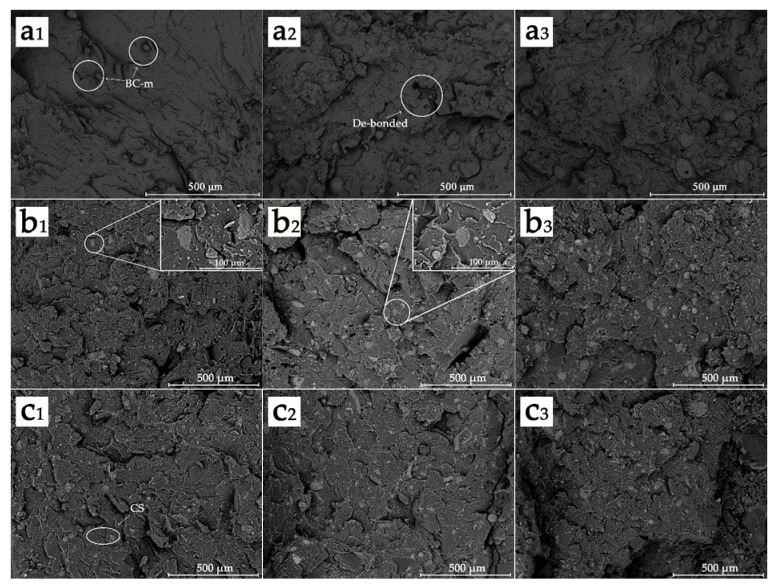
SEM images of fracture surface morphology: (**a_1_**) FPLA-B1, (**a_2_**) FPLA-B2, (**a_3_**) FPLA-B3, (**b_1_**) FPLA-C31, (**b_2_**) FPLA-C32, (**b_3_**) FPLA-C33, (**c_1_**) FPLA-C51, (**c_2_**) FPLA-C52, and (**c_3_**) FPLA-C53.

**Figure 4:**
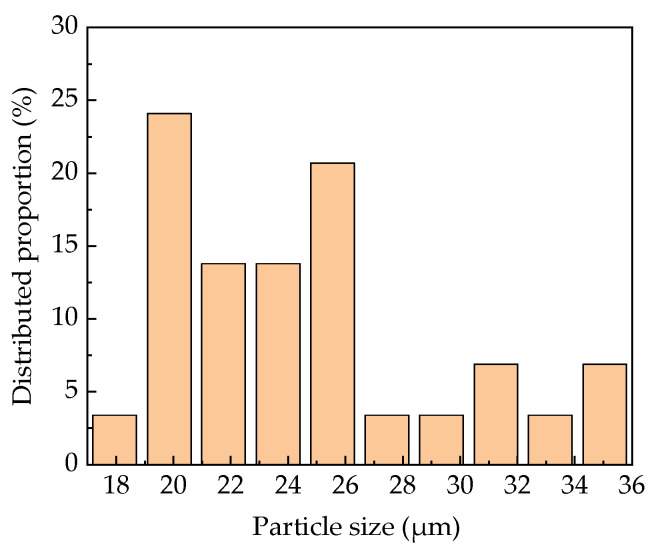
Typical particle size distribution of BC-m particles on fracture surface.

To investigate the dispersion in PLA, observation under SEM at low magnification (120×) was performed and the corresponding images are shown in Figure 3. Figure 3a_1_–3a_3_ show the fracture surface morphology with different BC-m addition levels indicating uniform distribution of BC-m in the polymer. At higher levels of BC-m (30%), the localized agglomeration of particles was evident (Figure 3a_2_) leading to a weak polymer-filler interface. The typical particle size of BC-m at the fracture surface was also analyzed, with results shown in Figure 4. A smaller particle size (mostly 20–26 μm) was observed due to cyclic extrusion of the screw during the melt compounding process.

The addition of CS (Figure 3b_1_,b_2_) improved the interfacial bonding, whereby no obvious delamination could be observed compared to if BC-m was added on its own. CS appears to facilitate BC-m dispersal and bonding with the PLA by acting as an interfacial adhesive.

#### 3.1.2. Thermal Properties

**Figure 5:**
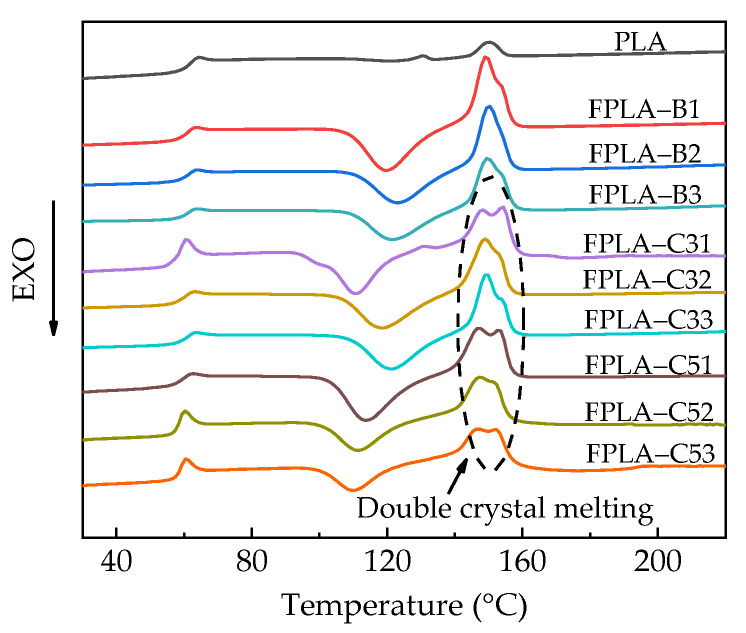
Heat flow curves of PLA and its blends.

To investigate the thermodynamic properties of PLA and its blends, differential scanning calorimetry (DSC) was carried out with heat flow curves shown in Figure 5. Key data, such as glass transition temperature (T_g_), cold crystallization temperature (T_cc_), crystallization enthalpy (ΔH_c_), melting temperature (T_m_), and melting enthalpy (ΔH_m_) are summarized in Table 2. 

From Table 2, the T_g_ values decreased with the addition of BC and CS, likely caused by increased plasticization of the polymer [35,39]. At a particular level of BC-m (e.g., 20% as bold highlighted) T_g_ values were minimally affected by CS level but *X*_c_ increased. Higher molecular mobility also means that more energy is needed to achieve crystallization while weakening the capacity for non-isothermal crystallization [30,39], leading to an increase in *T*_cc_ by 15.1 °C at most. The nucleation by the fillers contributed greatly to the improvement of *X*_c_. With the increasing addition of BC and CS, the *X*_c_ of composites increased from 0.9% to over 29%, the highest being FPLA-C33 with *X*_c_ = 38.92%, or 43 times that of pure PLA. The improvement of crystallization behavior also means that the thermal stability of the composite may be enhanced. Double melt crystallization peaks appeared with the addition of CS (Figure 5), especially at 3 wt.%, which may be attributed to incomplete cold crystallization at low temperatures resulting in the melting and recrystallization of α phase [17]. Equally, a slight decrease in *T*_m_ was observed with increase of CS content, as melting involves the same molecular movement condition [39]. The crystallization results suggest CS may play an important bridging role between BC-m and PLA by reducing the thickness and volume of the crystalline polymer interface [17].

#### 3.1.3. X-ray Diffraction Analysis

**Figure 6:**
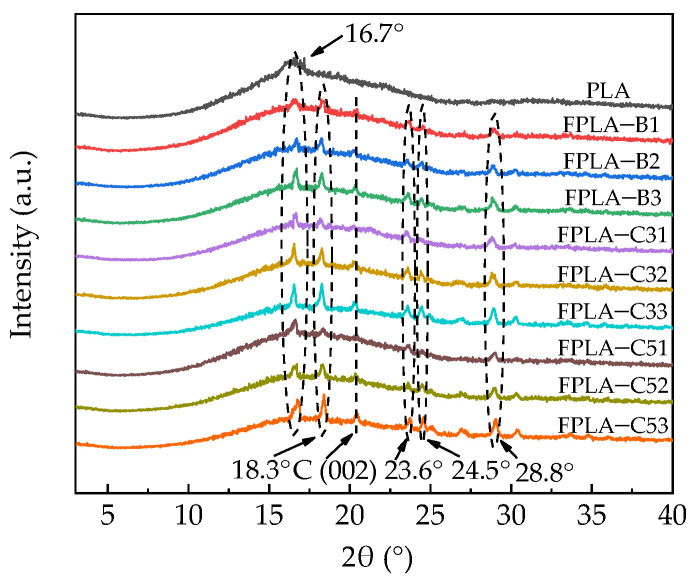
Diffraction patterns of PLA and FPLA composites.

X-ray diffraction was used to detect the differences in crystalline structure between PLA and FPLA composites (Figure 6). Only a broad diffraction peak for crystallinity in pure PLA at 16.7° was recorded, assigned to the α-phase crystallite [40,41]. Generally, the crystallization of pure PLA occurs too slowly to register any significant crystallinity, especially under the non-isothermal conditions encountered during extrusion and injection molding [41]. In contrast, a sharp crystallization peak at 16.7° appeared in FPLA-CS, consistent with DSC results. With the addition of BC-m and chitosan in different ratios, several new diffraction peaks at 2θ = 18.3°, 20.9°, 23.6°, 24.5°, and 28.8° appeared in the XRD curve. As reported previously, the peak at 2θ = 20.9° belongs to the graphitized structure of BC [17], and the 2θ = 24.5° and 28.8°corresponds to the β-form crystal structure [42]. The peak values of all FPLA materials increased and became sharp at 16.7°, which indicates the crystallinity index of PLA increased [43]. New peaks at 2θ = 18.3° and 23.6° suggest reorganization of the crystal structure [44]. 

### 3.2. Mechanical Properties

**Figure 7:**
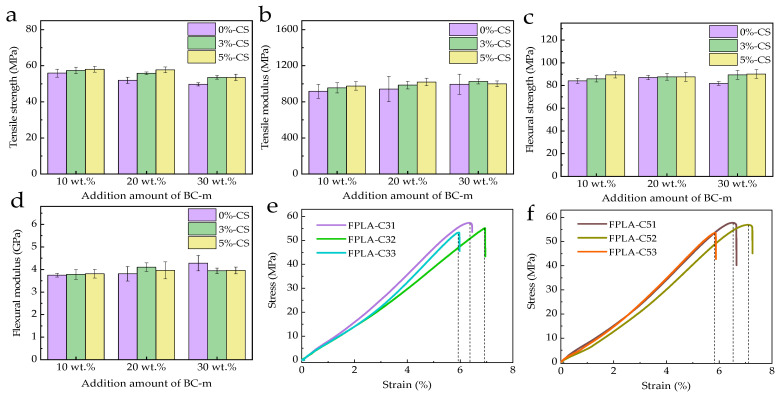
Average mechanical properties of FPLA-B and FPLA-C composites: (**a**) tensile strength, (**b**) tensile modulus, (**c**) flexural strength, and (**d**) flexural modulus; as well as typical tensile stress-strain curves of a selected sample: (**e**) FPLA-B composites and (**f**) FPLA-C composites.

Figure 7 shows the tensile and flexural properties of the composites with different BC-m and CS addition levels, while the detailed data are listed in Table 3.

As noted in previous work [18], the tensile strength of FPLA-B composites decreases with increased BC-m addition, with a 11.09% decrease at 30 wt.% BC-m. Adding CS significantly improved the tensile properties as seen in Figure 7a,b and in Table 3 bold values of FPLA-B composites. CS at 5 wt.% produced the largest increases: 11.11% in tensile strength and 8.42% in tensile modulus. Flexural properties were less changed by CS addition (Figure 7c,d, Table 3). Typical stress-strain curves for CS-added FPLA-Cs are shown in Figure 7e,f. Note the elongation at break is more sensitive to the compatibilization effect in multicomponent blends compared with other mechanical properties [45]. Adding 20 wt.% BC-m and 3 wt.% or 5 wt.% CS produced a more ductile composite with elongation at the break reaching 6.94% and 7.08%, respectively, which is 2.20% and 8.59% higher than that of 10% BC-m added with same content of CS.

CS particles with medium viscosity are dispersed in advance forming a flexible membrane locally under the action of pressure and temperature and effectively promoting the interfacial bonding between the filler and matrix, as indicated through observation of fracture morphology and higher composite strength and the modulus results. The enhancement is limited to low doses of CS as it is strongly hydrophilic due to –OH and –NH_2_ groups along the chain [46], whereas PLA is strongly hydrophobic [47,48]. As CS loading increases, the extent of interfacial zones with different affinities leads to a weakening in interfacial adhesion and interaction [28].

### 3.3. Thermal Degradation Behavior

#### 3.3.1. TG in N_2_

**Figure 8:**
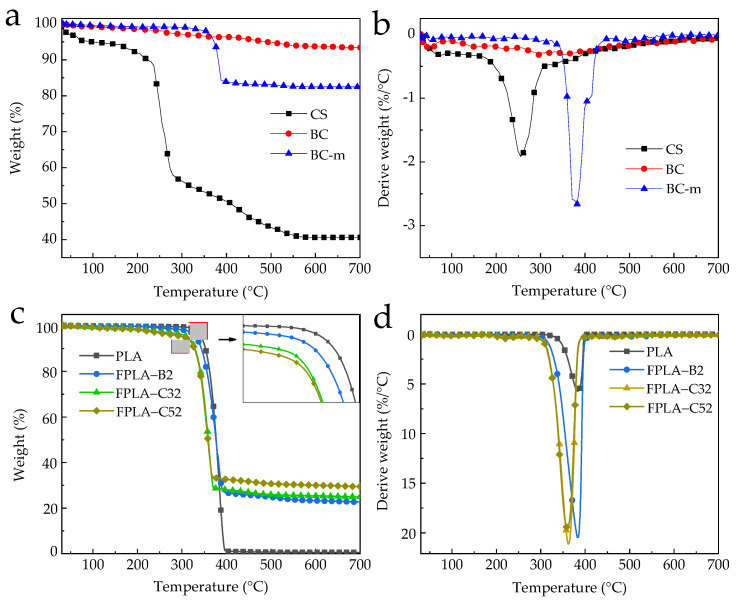
Typical TGA (**a**,**c**) and DTG (**b**,**d**) curves of CS, BC; BC-m, PLA, and FPLA composites in N_2_.

Selected TG and DTG curves of PLA, PLA/20BC-m, PLA/20BC-m/3CS, and PLA/20BC-m/5CS in nitrogen atmosphere are shown in Figure 8, and corresponding data for all mixes are shown in Table 4. The onset degradation temperature of samples is defined as the temperature at 5% weight loss (*T*_−5%_), and *T*_max_. is the temperature at the maximum rate of rate of mass loss.

Typical TG and DTG spectra for the constituents CS, BC, and BC-m under nitrogen (N_2_) atmosphere are shown in Figure 8a,b. CS started to thermally decompose at 103 °C. R_peak_ occurred at 255 °C and the final carbon residue at 700 °C was 40.60%. In contrast, BC showed excellent thermal stability with almost no significant mass loss during pyrolysis. R_peak_ was 0.32% at 295 °C and the final carbon residue was 93.39%. BC-m underwent rapid mass loss of its graft products at 382 °C and with a final carbon residue of 82.47%. The grafted phosphorous and nitrogen compounds on BC-m reduced the relative mass of pure BC in the filler by 10.94%.

Data for FPLA composites are shown in Figure 8c,d and Table 3, with notable contrasts for 20%BC-m with or without CS in bold highlight. FPLA composites were characterized by one *T*_max_ and *T*_-5%_ values were lower than for pure PLA, especially with CS addition. This is related to the early thermal decomposition of BC-m graft products in keeping with thermal degradation of IFRs [49,50] and contributing to its flame retardancy. The residue mass (RM) values of all FPLA were higher than pure PLA (0.60%) at 700 °C. The RM values of CS-added materials at the same level of BC-m content were higher again, suggesting a cooperative effect. This is evidenced by the RM being higher than the theoretical value for BC-m composites above 20% BC-m. The RM of FPLA-C52 (20%BC-m+5%CS) for example, was 29.42%, i.e., 55.1% higher than its theoretical value of 18.97%. BC-m decomposition produces phosphoric acid (PA) which accelerates the degradation of PLA in cooperation with higher content of CS further reducing *T*_-5%_ and earlier char formation, potentially providing enhanced combustion resistance [21,33,34]. Note the increase in RM is limited at low (3%) CS addition level.

#### 3.3.2. TG in Air

**Figure 9:**
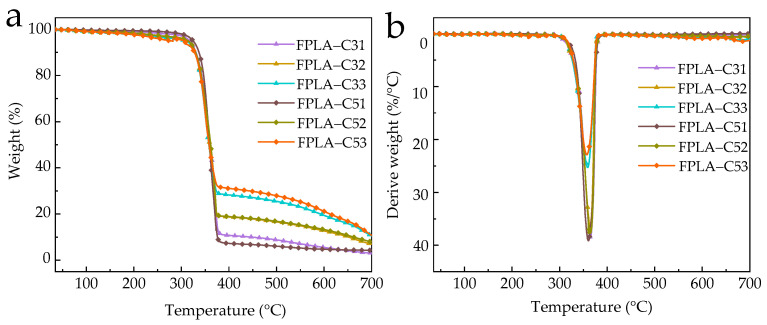
TGA (**a**) and DTG (**b**) curves of FPLA composites in air.

TG results in air are given in Figure 9 and Table 5.

Compared with nitrogen atmosphere, T_-5%_ values increased that with 5 wt.% CS and T_-5%_ values of FPLA-C51, FPLA-C52, and FPLA-C53 increased by 12 °C, 7 °C, and 3 °C, respectively. All values for R_peak_ of the FPLA composites rose as well, while corresponding T_max_ values decreased. Oxygen promotes the pyrolysis process of PLA by oxidizing the molecular chains [10], intensifying the pyrolysis reaction and promoting the formation of the carbonized char layer, resulting in earlier inhibition of mass loss. TGA results in air still indicate adding BC-m and CS as a cooperative system is effective in promoting the early formation of char residue but note here the much smaller difference between CS levels when added to a base level of 20%BC-m (bold in Table 5).

### 3.4. Combustion Characterization

#### 3.4.1. LOI and UL-94

**Figure 10:**
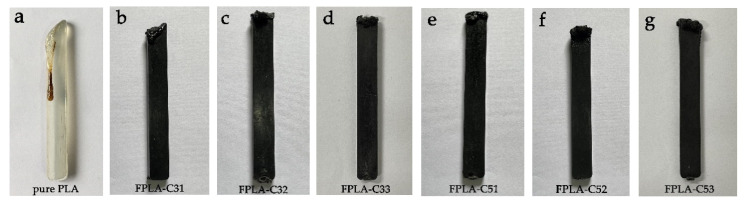
Digital images of PLA and FPLA composites after LOI tests: (**a**) pure PLA; (**b**) 3CS/10BC-m/87PLA; (**c**) 3CS/20BC-m/77PLA; (**d**) 3CS/30BC-m/67PLA; (**e**) 5CS/10BC-m/85PLA; (**f**) 5CS/20BC-m/75PLA; (**g**) 5CS/30BC-m/65PLA.

Flammability indices for PLA and FPLA composites (LOI and UL-94 tests) are summarized in Table 6 with contrasts for CS level with 20%BC-m in bold. Images of PLA and FPLA-C composites after the LOI tests are shown in Figure 10.

Pure PLA burns very easily and exhibited obvious melt dripping (Figure 10a) with a LOI of 20.7 vol%. CS has little flame retardancy if added alone; LOI values were increased slightly to 21.5 vol% and 21.7 vol%, respectively. Our previous findings [18] showed that with 20 and 30 wt.% addition of BC-m, LOI increased to 29.2 vol% and 32.1 vol%, respectively, and melt dripping was completely suppressed, achieving a UL-94 V-0 rating. However, 10 wt.% BC-m was insufficient to prevent melting [18]. With co- addition of CS, the expansion phenomenon at the burning tip became more obvious, as seen in Figure 10d,g. CS addition further increased the LOI of 20% BC-m from 29.2% vol% to 31.3 vol% and 32.4 vol% for the 3% and 5% CS addition, respectively.

The possible cooperative effect of adding BC-m and CS in combination was further explored using the Lewin and Weil concept of cooperative effectiveness (Ce) [51], calculated as follows [52]:

Ce = {(F_p_)_[fr__+s]_ − (F_p_)_P_}/{(F_p_)_fr_ − F_p_)_P_) + ((F_p_)_s_ − (F_p_)_P_} (2)

where (F_p_)_P_ is the flame-retardant property of the polymer alone, (F_p_)_fr_ is the polymer containing BC-m, (F_p_)_s_ is the polymer with CS, and (F_p_)_[fr__+s]_ is the polymer with cooperative filler system (BC-m+CS). Cooperative behavior of the additives is considered to occur with the combination of BC-m and CS if Ce > 1. Since the LOI values are usually used to evaluate combustion intensity and directly correspond with UL-94 and cone calorimetry indices, LOI values were used for F_p_ in Equation (2) [16,52]. Ce values were above 1 for BC-m+CS composites (Table 6) suggesting a cooperative effect. Overall, 20 wt.% BC-m with 5% CS was the most effective, in accord with the TGA results under N_2_ atmosphere.

#### 3.4.2. Cone Calorimeter (CONE) Results

**Figure 11:**
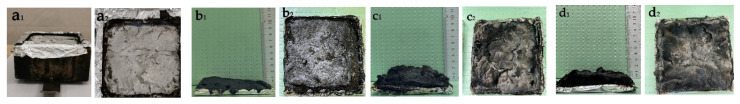
Images of carbon residues of PLA and FPLA composites after CONE tests: (**a_1_,a_2_**) PLA, (**b_1_,b_2_**) FPLA-B2, (**c_1_,c_2_**) FPLA-C32, and (**d_1_,d_2_**) FPLA-C52.

**Figure 12:**
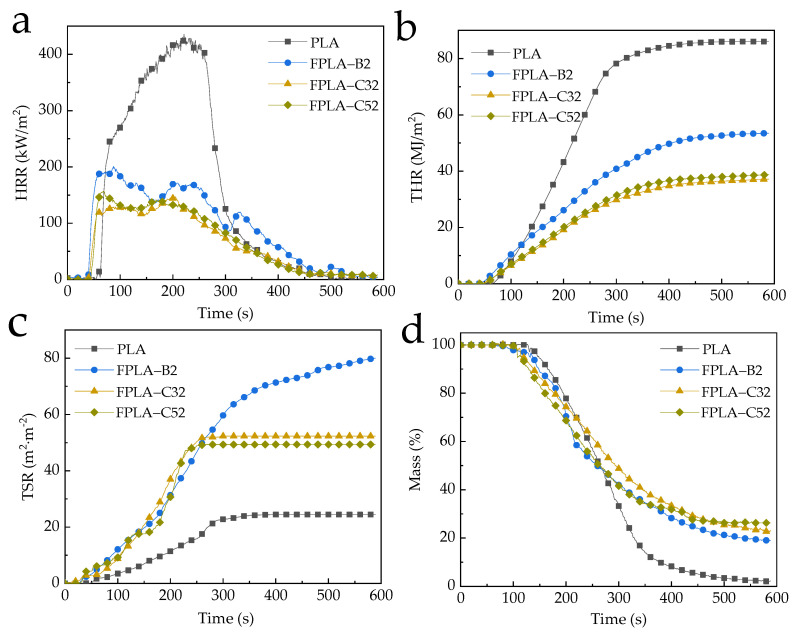
Typical CONE test results of PLA and FPLA composites: (**a**) HRR, (**b**) THR, (**c**) TSR, and (**d**) mass.

The appearance of char residues aids interpretation of CONE data [53]. Examples of chars from PLA and FPLA-C composites (at baseline 20% BC-m addition) after CONE tests are shown in Figure 11. With CS addition, the ‘puffiness’ of the char increased, as seen in Figure 11c_2_,d_2_. Key parameters include the heat release rate (HRR), total heat release (THR), total smoke release (TSR), residue mass (RM), and average effective heat of combustion (aEHC); with data for all samples given in Table 7. Selected curves for PLA, PLA+20%BC-m and this baseline with CS addition are shown for easier visual comparison in Figure 12.

HRR curves of selected samples are shown in Figure 12a, which a main factor indicating fire spread risk [54]. Contrasted CONE indices for PLA, PLA+20%BC-m and this baseline plus 3 or 5% CS addition are in bold in Table 7. The heat release of all specimens mainly occurred between 100 and 300 s. Peak heat release rate (pHRR) of pure PLA reached 435.58 kW/m^2^ and was reduced by just 4.01% when 10 wt.% BC-m was added [18]. Even at low BC-m addition, CS can be effective. From Table 7, co-adding 3 wt.% CS to this mix reduced pHRR to 216.33 kW/m^2^, a decrease of 50.34%. Adding 5 wt.% CS did not further limit heat release; in fact, for PLA/20%BC-m, adding 5 wt.% CS increased pHRR to 156.45 kW/m^2^ from 146.97 kW/m^2^ for 3% CS. CS addition significantly reduced THR and aEHC indices compared with PLA and PLA/20%BC-m (Figure 12b). 

From Table 7 and Figure 12a,b pHRR and THR for the baseline PLA+20%BC-m were further reduced by CS addition, particularly 3%. For example pHRR was reduced by almost 27% by adding 3%CS to the baseline PLA+20% BC-m. Note from Figure 12c CS addition curtailed TSR after about 250s compared with adding BC-m on its own. It seems likely that the cooperation of CS and BC-m accelerates the development of expanded char and greater amounts of non-flammable gases with heating, producing a greater peak mass loss rate (pMLR) and TSR. The char barrier is effective and works by blocking heat and oxygen, as shown in the reductions in pHRR and THR [55] when CS was added to all levels of BC-m addition. 

### 3.5. Residue Analysis

#### 3.5.1. Morphology of Residue Surfaces and Inner Layers

**Figure 13:**
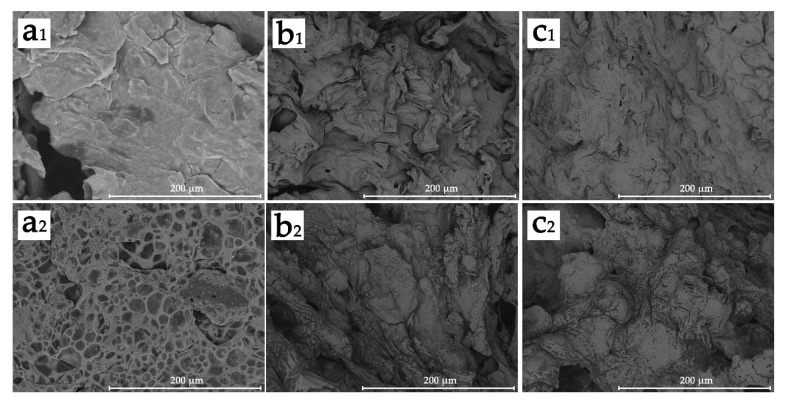
SEM images of residues on outer surface ((**a_1_**): FPLA-B3; (**b_1_**): FPLA-C33; (**c_1_**): FPLA-C53) and internal surface ((**a_2_**): FPLA-B3; (**b_2_**): FPLA-C33; (**c_2_**): FPLA-C53) from FPLA composites after cone tests.

The char residue on the outer and inner surface of FPLA-B and FPLA-C composites after CONE tests was analyzed by SEM, and the results are shown in Figure 13. When CS was not added, there was a dense char on the surface of the residue but with cavities (Figure 13a_1_), allowing heat and oxygen through. Adding CS helped produce a more continuous layer of char, as reflecting in the improved CONE test indices. Images of the internal char layer revealed changed morphology of the char layer. From previous work [18], PLA/BC-m, especially at 30% addition formed a distinct filled-pore structure shown in Figure 13a_2_. With CS addition the char was denser and well consolidated without bubbles, as shown in Figure 13b_2_,c_2_. An enhanced barrier role appears to be achieved through the cooperative effect of CS and incombustible gases and acids produced by the thermal decomposition of BC-m. Adding BC-m to PLA causes pyrophosphate products to fill the pores in the char produced by gases generated during the thermal degradation of BC-m graft products [18]. As combustion progresses, flame suppression transitions to the condensed phase protective mechanism.

#### 3.5.2. Structure and Composition

**Figure 14:**
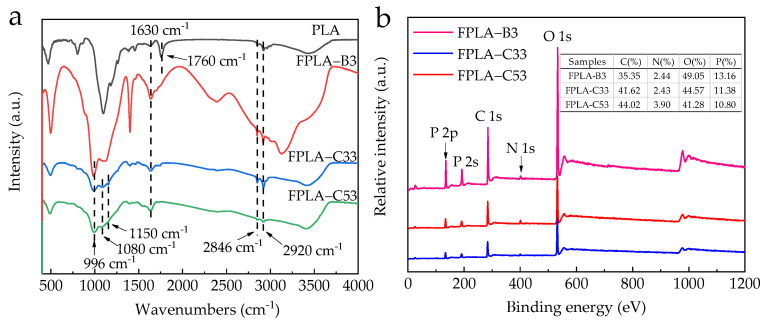
(**a**) FTIR curves and (**b**) XPS spectra of the residue char for PLA, FPLA-B3, FPLA-C33, and FPLA-53.

In order to elucidate differences in the char residue structure, the residues of PLA, FPLA-B3, FPLA-C33, and FPLA-C53 (maximum level of BC-m addition) were analyzed by FTIR, with results shown in Figure 14a. The typical characteristic peaks of PLA at 1630 cm^−1^, 1760 cm^−1^, 2846 cm^−1^, and 2920 cm^−1^ were identified, corresponding to C=C, C=O, and symmetrical and asymmetrical stretching of –CH_2_– groups, respectively [56,57]. Adding BC-m and CS together produced significantly different spectra compared with PLA, with new absorption peaks at 996 cm^−1^, 1080 cm^−1^, and 1168 cm^−1^ in FPLA-C33 and FPLA-C53. These correspond to the stretching vibration of P=O, P–O–C, and P–O–P [56,58] from the graft products on BC-m, indicating pyrophosphates that contribute to the formation of char residue [56].

To further investigate the chemical element components of the char residues after CONE testing, XPS was performed with the corresponding spectra shown in Figure 14b. C and O contents exceeded 35%, making up the majority of the residue. C content increased with greater CS addition.

**Figure 15:**
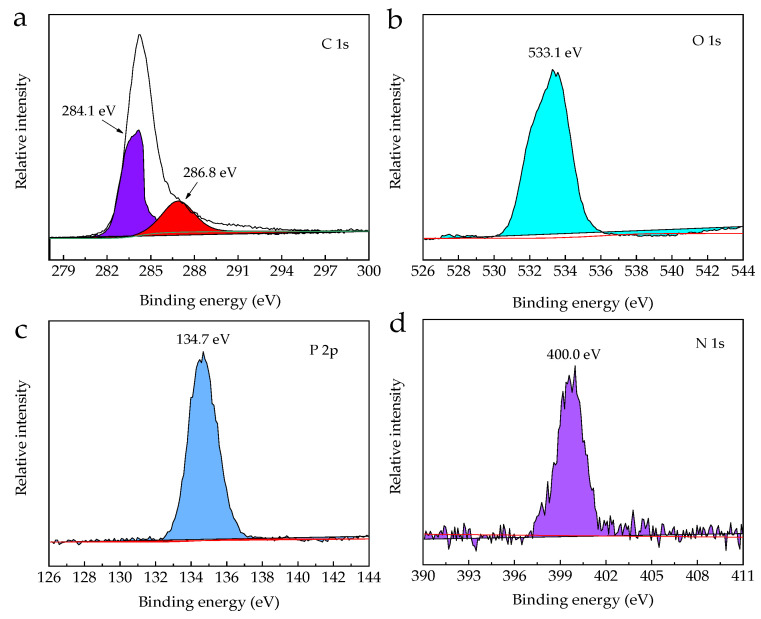
Spectra for char residues of FPLA-C53: (**a**) C 1s, (**b**) O 1s, (**c**) P 2p, and (**d**) N 1s.

Specific spectra are shown in Figure 15 for elements on the surface of the carbon residue of FPLA-C53. From Figure 15a, the binding energy (BE) at 284.1 eV and 286.8 eV for C 1s peaks was assigned to C–C or C=C groups in aliphatic and aromatic fragments, or C–O in P–O–C groups, respectively, while the signal at 533.1 eV in Figure 15b corresponds to –O– in P–O–C [59]. The peak at 134.7 eV of the P 2p spectra in Figure 15c and 401.2 eV of N 1s spectra in Figure 15d correspond to the P–O–P bond and the C–N or C=N in the triazine structures, respectively [16,59,60]. Theoretically, the content of elemental C in FPLA-C33 and FPLA-C53 should be increased by 3% and 5% above that of the 30%BC-m addition, whereas the actual C values in the residue of these composites were 41.62% and 44.02%, respectively. A higher percentage of elemental C means that more organic matter in the composite remained after combustion testing. The results add further evidence that CS behaves cooperatively with BC-m dispersed in the PLA matrix leading to the formation of a denser and more stable expanded char layer structure containing P and N reflected in the detection of C–O–P, P–O–P, and C–N.

## 4. Conclusions

(1) Co-adding 3 or 5 wt.% CS with BC-m to PLA enhanced tensile properties of PLA. CS is an interfacial adhesion promoter which improves the interfacial compatibility between BC-m and PLA. Adding 3 wt.% CS and 30 wt.% BC-m increased the crystallinity to 38.92% or 43 times that of pure PLA (0.9%). The internal crystal structure was reorganized by CS addition and the crystallinity index was increased.
(2) TGA showed early thermal degradation of CS-added PLA/BC-m composites and enhanced residue mass. In nitrogen atmosphere co-adding 5 wt.% CS and 20 wt.% BC-m, the corresponding residue (29.4%) was 55.1% higher than the theoretical value (18.97%).
(3) Co-adding 5 wt.% CS and 30 wt.% BC-m, increased LOI to 33.6 vol%; 4.7% higher than adding BC-m alone. UL-94 V-0 rating at low BC-m addition (10 wt.%) was improved correspondingly. Cooperative effectiveness analysis (Ce) on LOI results suggest that active cooperation between CS and BC-m improves the flame retardancy of PLA/BC-m composites.
(4) CONE results showed very effective further combustion suppression with just 3% CS co-addition to the effective baseline of 20% or more BC-m addition. For example peak heat release rate (pHRR) was reduced by almost 27% for 3% CS + 20% BC-m, and total heat release (THR) was reduced by 30.5%. Analysis of char morphology, chemical elements, and structure reveals that CS and BC-m form a denser, more stable carbonized layer that is effective in heat and oxygen insulation, resulting in improved flame retardancy of PLA composites.
(5) A mix of just 20 wt.% BC-m + 3 wt.% CS may be a viable flame-retardant system that meets the requirements for a non-toxic, strong and biodegradable PLA packaging product.

## Figures and Tables

**Table 1:** Formulations for PLA and its blends.

Scheme 100	Composition (wt.%)		
	PLA	BC-m	CS
PLA	100	0	0
FPLA-C3	97	0	3
FPLA-C5	95	0	5
FPLA-B1	90	10	0
FPLA-B2	80	20	0
FPLA-B3	70	30	0
FPLA-C31	87	10	3
FPLA-C32	77	20	3
FPLA-C33	67	30	3
FPLA-C51	85	10	5
FPLA-C52	75	20	5
FPLA-C53	65	30	5

**Table 2:** DSC indices for PLA and FPLA composites.

Sample	*T*_g_ (°C)	*T*_cc_ (°C)	*T*_m1_ (°C)	*T*_m2_ (°C)	Δ*H*_c_ (J/g)	Δ*H*_m_ (J/g)	*X*c (%)
**PLA**	**59.1**	**108.1**	**150.8**	**-**	**0.84**	**4.06**	**0.90**
FPLA-B1	58.1	119.8	149.2	-	27.39	27.77	32.51
**FPLA-B2**	**58.4**	**123.2**	**150.1**	**-**	**22.27**	**22.87**	**29.74**
FPLA-B3	58.7	121.6	149.7	-	21.26	20.71	32.45
FPLA-C31	56.9	110.8	148.2	154.2	27.19	20.98	33.39
**FPLA-C32**	**57.1**	**118.4**	**149.0**	**-**	**25.63**	**24.44**	**35.56**
FPLA-C33	58.1	121.1	149.3	-	24.41	23.36	38.92
FPLA-C51	57.1	114.0	147.2	152.9	30.43	28.8	38.25
**FPLA-C52**	**57.2**	**120.4**	**148.7**	**154.0**	**25.91**	**25.18**	**36.91**
FPLA-C53	57.6	112.1	145.1	152.6	18.99	18.16	31.21

**Table 3:** Mechanical properties of the FPLA-B and FPLA-C composites.

Sample	Tensile Strength (MPa)	Tensile Modulus (MPa)	Flexural Strength (MPa)	Flexural Modulus (GPa)
**^a^ PLA**	**58.12 ± 1.76**	**774.24 ± 33.43**	**95.76 ± 0.80**	**3.73 ± 0.17**
^a^ FPLA-B1	55.91 ± 2.23	917.06 ± 77.74	84.15 ± 2.18	3.75 ± 0.08
**^a^ FPLA-B2**	**51.94 ± 1.77**	**941.15 ±141.33**	**87.01 ± 1.90**	**3.81 ± 0.32**
^a^ FPLA-B3	49.71 ± 0.88	995.70 ± 112.20	81.77 ± 1.65	4.28 ± 0.34
FPLA-C31	57.40 ± 1.83	955.02 ± 56.03	85.95 ± 2.84	3.78 ± 0.21
**FPLA-C32**	**55.84 ± 0.67**	**985.16 ± 42.75**	**87.70 ± 2.94**	**4.10 ± 0.19**
FPLA-C33	53.56 ± 1.02	1026.41 ± 27.41	89.37 ± 3.94	3.94 ± 0.12
FPLA-C51	58.00 ± 1.61	976.00 ± 47.30	89.40 ± 2.68	3.81 ± 0.19
**FPLA-C52**	**57.71 ± 1.57**	**1020.43 ± 41.14**	**87.55 ± 3.90**	**3.96 ± 0.38**
FPLA-C53	53.54 ± 1.77	999.86 ± 32.44	90.12 ± 3.87	3.95 ± 0.14

Notes: ^a^ Reprinted with permission from ref [18]. Copyright 2021 American Chemical Society. Copyright Liang Zhang, Weisheng Chai, Wenzhu Li, Kate Semple, Ningning Yin, Wenbiao Zhang, Chunping Dai.

**Table 4:** TGA data of PLA and FPLA composites in N_2_.

Samples	T_-5%_ (°C)	R_peak_/T_max_ (%·min^−1^/°C)	Residue Mass (%)
			700 (°C)
BC	484	0.32/295	93.39
BC-m	373	2.66/382	82.47
CS	103	1.92/255	40.60
**PLA**	**343**	**5.72/382**	**0.60**
^a^ FPLA-B1	334	25.33/385	8.64
**^a^ FPLA-B2**	**331**	**20.94/383**	**21.81**
^a^ FPLA-B3	331	19.52/380	29.83
FPLA-C31	316	22.24/365	8.97
**FPLA-C32**	**314**	**21.14/362**	**24.92**
FPLA-C33	311	18.81/359	29.56
FPLA-C51	312	23.46/365	9.64
**FPLA-C52**	**306**	**19.55/360**	**29.42**
FPLA-C53	304	18.65/356	32.74

Notes: ^a^ Reprinted with permission from ref [18]. Copyright 2021 American Chemical Society. Copyright Liang Zhang, Weisheng Chai, Wenzhu Li, Kate Semple, Ningning Yin, Wenbiao Zhang, Chunping Dai.

**Table 5:** TGA data of FPLA composites under air condition.

Samples	T_-5%_ (°C)	R_peak_/T_max_ (%·min^−1^/°C)	Residue Mass (%)
			700 (°C)
FPLA-C31	317	38.48/363	3.01
**FPLA-C32**	**314**	**36.57/362**	**6.99**
FPLA-C33	311	25.30/358	10.48
FPLA-C51	324	39.14/360	4.30
**FPLA-C52**	**313**	**37.76/360**	**8.10**
FPLA-C53	307	22.90/355	10.70

**Table 6:** LOI and UL-94 results of PLA and FPLA composites.

Samples	LOI ± 0.2 (vol%)	UL-94			Ce
		^a^ t_1_ (s)/t_2_ (s)	Dripping	Rating	
PLA	20.7	^b^ TB	Yes	^c^ NR	-
FPLA-C3	21.5	TB	Yes	NR	-
FPLA-C5	21.7	TB	Yes	NR	-
^d^ FPLA-B1	28.0	5.6/2.3	Yes	V-2	-
^d^ FPLA-B2	29.2	2.1/1.1	No	V-0	-
^d^ FPLA-B3	32.1	1.2/1.4	No	V-0	-
FPLA-C31	29.0	4.7/2.0	No	V-1	1.02
**FPLA-C32**	**31.3**	**1.9/1.1**	**No**	**V-0**	**1.14**
FPLA-C33	33.0	1.2/1.3	No	V-0	1.01
FPLA-C51	29.2	4.6/2.1	No	V-1	1.02
**FPLA-C52**	**32.4**	**1.8/1.2**	**No**	**V-0**	**1.23**
FPLA-C53	33.6	1.0/1.0	No	V-0	1.04

^a^ average combustion times after the first and second applications of the flame; ^b^ TB = totally burnt down to clamp; ^c^ No rating; ^d^ Reprinted with permission from ref [18]. Copyright 2021 American Chemical Society. Copyright Liang Zhang, Weisheng Chai, Wenzhu Li, Kate Semple, Ningning Yin, Wenbiao Zhang, Chunping Dai.

**Table 7:** ^a^ Cone calorimeter results for each sample at 35 kW/m^2^.

Samples	TTI (s)	pHRR (kW/m^2^)	THR (MJ/m^2^)	pMLR (g/s)	aEHC (MJ/kg)	RM (%)
**PLA**	**56**	**435.58**	**86.06**	**0.60**	**16.37**	**2.29**
^b^ FPLA-B1	34	418.11	68.64	0.51	16.15	6.45
**^b^ FPLA-B2**	**33**	**201.02**	**53.48**	**0.48**	**15.08**	**18.99**
^b^ FPLA-B3	31	181.61	41.10	0.35	14.52	47.71
FPLA-C31	37	216.33	54.19	0.67	12.28	8.36
**FPLA-C32**	**35**	**146.97**	**37.17**	**0.61**	**12.04**	**23.17**
FPLA-C33	34	123.38	29.00	0.37	11.01	35.99
FPLA-C51	36	268.56	42.04	0.52	12.68	8.22
**FPLA-C52**	**37**	**156.85**	**38.79**	**0.74**	**11.67**	**26.26**
FPLA-C53	34	125.49	25.65	0.53	10.66	38.18

Notes: ^a^ TTI, time to ignition, ±2 s; pHRR, peak heat release rate, ±15 kW/m^2^; pMLR, peak mass loss rate, ±0.05 g/s; aEHC, average effective heat of combustion within 180 s, ±2 MJ/kg; ^b^ Reprinted with permission from ref [18]. Copyright 2021 American Chemical Society. Copyright Liang Zhang, Weisheng Chai, Wenzhu Li, Kate Semple, Ningning Yin, Wenbiao Zhang, Chunping Dai.

## Data Availability

The processed data required to reproduce these findings cannot be shared at this time since the data also contribute to part of an ongoing study.
